# Vancomycin-associated linear IgA disease mimicking toxic epidermal necrolysis*

**DOI:** 10.1590/abd1806-4841.20164665

**Published:** 2016

**Authors:** Amanda Regio Pereira, Luis Henrique Barbizan de Moura, Jhonatan Rafael Siqueira Pinheiro, Victor Pavan Pasin, Milvia Maria Simões e Silva Enokihara, Adriana Maria Porro

**Affiliations:** 1 Universidade Federal de São Paulo (Unifesp) - São Paulo (SP), Brazil

**Keywords:** Linear IgA bullous dermatosis, Drug eruptions, Vancomycin

## Abstract

Linear IgA dermatosis is a rare subepidermal autoimmune blistering disease characterized by linear deposition of IgA along the basement membrane zone. In the last three decades, many different drugs have been associated with the drug-induced form of the disease, especially vancomycin. We report a case of vancomycin-induced linear IgA disease mimicking toxic epidermal necrolysis. The aim of this work is to emphasize the need to include this differential diagnosis in cases of epidermal detachment and to review the literature on the subject and this specific clinical presentation.

## INTRODUCTION

Linear IgA dermatosis (LAD) is a rare autoimmune mucocutaneous blistering disease characterized immunohistopathologically by subepidermal blister and linear deposition of IgA along the basement membrane zone (BMZ) on direct immunofluorescence (DIF). It is classified as spontaneous and drug-induced (DI-LAD) forms.^1^ Since the first publication about DI-LAD in 1981, more than one hundred cases have been reported associating different drugs with the disease, especially vancomycin. ^2,3^

Annular or polycyclic plaques and papules with blistering around the edges (string of pearls sign) is the classic presentation of LAD, occurring usually in childhood.^4^ Development of LAD in adulthood can be clinically polymorphic, mimicking dermatitis herpetiformis (DH), bullous pemphigoid (BP), pemphigus vulgaris, erythema multiforme and toxic epidermal necrolysis (TEN).^5^

DI-LAD tends to be more severe, extensive and atypical than spontaneous LAD. In a recent study with an expressive number of cases for a rare condition, the frequencies of mucosal involvement, presence of target or target-like lesions and the string of pearls sign did not differ between the two forms of the disease. However, Nikolsky's sign and large erosions were significantly more frequent in patients with DI-LAD.^1^

Few cases have been published of the rare and dramatic TEN-like clinical presentation of the disease.^5^ This report is justified by its iconographic exuberance, rarity and the need to include the disease in the differential diagnosis of patients with epidermal detachment.

## CASE REPORT

**Figure 1:**
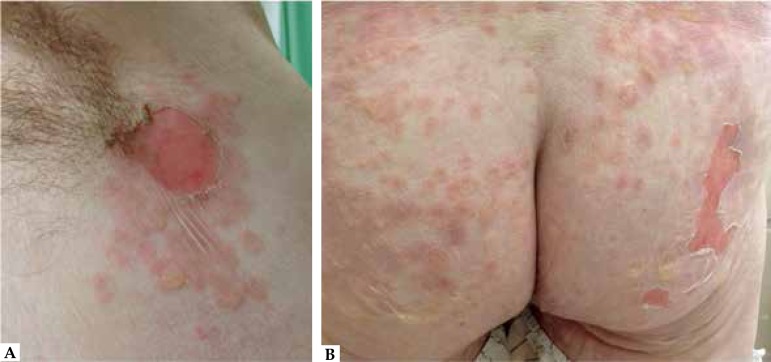
**A)** Axillary region with flaccid bullae and erosion. **B)** Confluent flaccid blisters and vesicles with erosions and epidermal detachment on the buttocks

**Figure 2:**
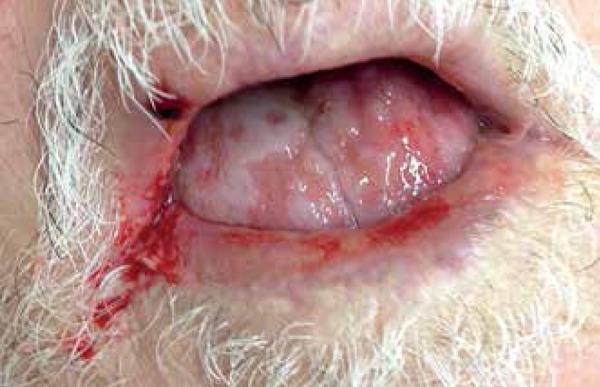
Extensive involvement of the oral mucosa and lips with erosions and friability

A 75-year-old male patient was hospitalized for surgical myocardial revascularization. Past medical history included high blood pressure, diabetes, dyslipidemia and ischemic stroke. In the previous three days, he had developed painful oral ulcers, tense and flaccid bullae and vesicles over the limbs and trunk. We also identified erosions on the buttocks and a positive Nikolsky's sign (Figures 1 and 2). Abrupt onset and rapid progression of the lesions were observed. The patient had been under empiric therapy with intravenous vancomycin and ceftriaxone since the day of the surgery, ten days before the lesions started to appear. The remaining drugs in use were enoxaparin, tramadol, amiodarone and medicines under continuous and chronic prescription (losartan, aspirin, propranolol, amlodipine, clonidine and simvastatin). The vancomycin level was 31.7 µg/ml, which is above the recommended therapeutic concentration (15-20 µg/ml).

**Figure 3:**
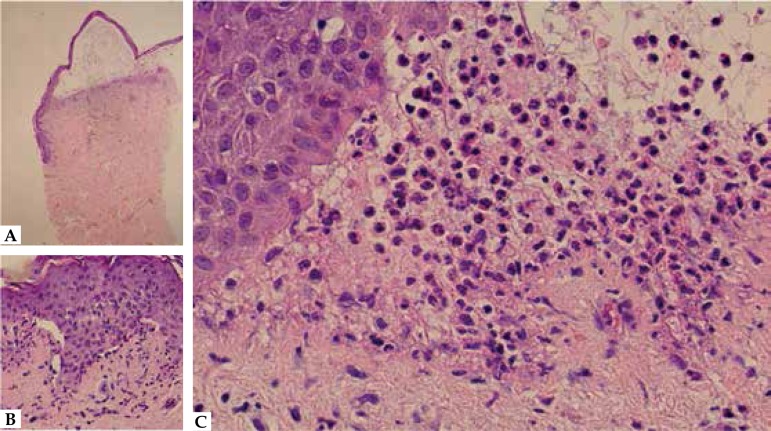
Histopathology of an intact vesicle (hematoxylin and eosin). **A** and **B)** Subepidermal cleavage – 40x and 400x magnification; **C)** Neutrophilic infiltrate – 400x magnification

**Figure 4:**
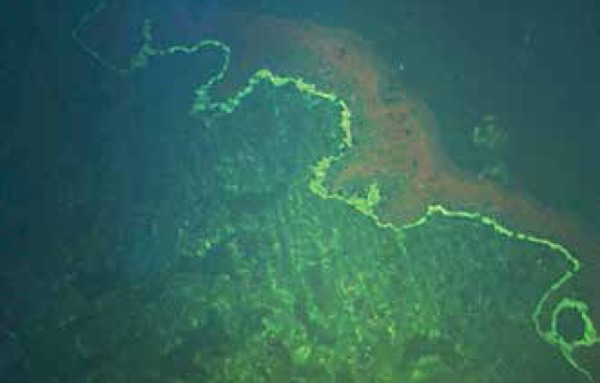
Direct immunofluorescence on perilesional skin revealing linear IgA deposition at the basement membrane zone

Considering the clinical picture and drug history, vancomycin-induced LAD was suspected. TEN and BP were considered in the differential diagnosis. Biopsy of an intact vesicle revealed subepidermal cleavage with neutrophilic infiltrate (Figure 3). DIF on perilesional skin demonstrated linear IgA deposition at the BMZ and negative IgG, IgM and C3 fluorescence (Figure 4).

New lesions stopped appearing eight days after antibiotic discontinuation and administration of prednisone 0.5 mg/Kg/d. Complete healing was observed after twelve days. However, the patient died of sepsis and respiratory failure two weeks after total reepithelization of the skin.

## DISCUSSION

Up to 2013, 16 cases of DI-LAD mimicking TEN had been reported. These cases were reviewed by Kakar *et al.*, who found an average age of 69 years, variable mucous involvement (8/13), positive Nikolsky's sign in 8 out of 10 cases and affected body surface area ranging from 15%-90%. All surviving patients showed resolution of lesions two or more weeks after discontinuation of the putative drug. Six deaths were not attributable to DI-LAD itself, as in our case. Treatment of DI-LAD usually consists of discontinuation of the offending medication. In this review, however, six patients were also treated with dapsone, corticosteroid or intravenous immunoglobulin.^5^

In the present report, chronology (onset of lesions 10 days after vancomycin introduction, and resolution 12 days after discontinuation) is compatible with the literature. In the cases associated with vancomycin reviewed by Fortuna *et al.,* latency period ranged from 2 to 21 days and clinical remission occurred 1 to 21 days after drug discontinuation.^3^

The DIF pattern presented by our patient is classic in LAD, but C3 and IgM deposits can also be found in association with IgA. LAD histopathology is not specific and may be similar to DH and BP. A subepidermal blister with neutrophilic infiltrate is the most common description, but the infiltrate can be eosinophilic, mimicking BP.^4^

Identification of the target antigens by immunoblotting has been performed in a few cases of DI-LAD. Different molecular weight proteins of the BMZ have been implicated (83-, 97-, 130-, 210-, 230-, 285- (LAD285), 180Kda; NC16a domain of BP180; type VII collagen; α3 subunit of laminin-332).^6,7^ Because of the multiple possible antigens involved, indirect immunofluorescence on salt-split skin can be positive on the epidermal side, dermal side or both.^4,8^

DI-LAD pathogenesis has not been completely elucidated yet. Implicated drugs can cause disruption of self-tolerance by a hapten-mediated process or by structurally modifying proteins at the BMZ. Triggering events such as infections may act as co-factors.^9^

In 2012, Fortuna *et al.* reviewed the literature in journals indexed in PubMed and selected 52 cases of DI-LAD. Out of the total, 46.2% were related to vancomycin and 53.8% to other substances, especially captopril, trimethoprim/sulfamethoxazole, phenytoin and diclofenac.^3^ Chart 1 contains an updated list of drugs associated with LAD.^1^

**Chart 1:** List of drugs associated with LAD in the literature up to 2015

Vancomycin	
Captopril	Penicillin G
Trimethoprim/Sulfamethoxazole	Interferon / interleucina 2
Phenytoin	Verapamil
Diclofenac	Vigabatrin
Amiodarone	Imipenem
Piroxicam	Ketoprofen
Naproxen	Carbamazepine
Acetaminophen	Amlodipine
Ceftriaxon	Candesartan/Eprosartan
Amoxicillin	Somatostatin
Atorvastatin	Buprenorphine
Lithium carbonate	Metronidazole
Gemcitabine	Moxifloxacin
Ampicilin	Sulfasalazine
Furosemide	Cefuroxime axetil
	Ampicillin/sulbactam

Adapted from: Chanal et al., 2013.^1^

Despite the considerable number of studies reporting association between LAD and medications, there is poor evidence of causality in the majority of them. Diagnosis usually relies on chronology and previously published cases. However, as these patients are often exposed to many concurrent medications, establishing a cause-effect relationship may be challenging. Challenge-de-challenge-rechallenge testing protocol should be the gold-standard procedure, but is not always feasible because of ethical and operational aspects.^3^ A reasonable option is the use of internationally accepted algorithms for causality assessment in adverse drug reactions (ADR), such as Naranjo algorithm (Chart 2).^10^

**Chart 2:** Naranjo algorithm for causality assessment between a drug and possible related adverse reactions

Questions	YES	NO	UNKNOWN
1. Are there previous conclusive reports on this reaction?	+1	0	0
2. Did the adverse event appear after the suspected drug was given?	+2	-1	0
3. Did the adverse reaction improve when the drug was discontinued	+1	0	0
or a specific antagonist was given?			
4. Did the adverse reaction appear when the drug was readministered?	+2	-1	0
5. Are there alternative causes that could have caused the reaction?	-1	+2	0
6. Did the reaction reappear when a placebo was given?	-1	+1	0
7. Was the drug detected in any body fluid in toxic concentrations?	+1	0	0
8. Was the reaction more severe when the dose was increased or less	+1	0	0
severe when the dose was decreased?			
9. Did the patient have a similar reaction to the same drug or similar	+1	0	0
drugs in any previous exposure?			
10. Was the adverse event confirmed by any objective evidence?	+1	0	0
Score: ≥9 = definite adverse drug reaction (ADR); 5-8 = probable ADR; 1-4: possible ADR; 0 = doubtful ADR.			

Adapted from: Naranjo et al., 1981.^10^

In the case we presented, vancomycin was considered to have induced LAD due to chronological plausibility, a Naranjo score of 4 (possible ADR) and previous conclusive reports on this reaction.

This report aims to draw attention to the possibility of LAD in cases of suspected TEN and to emphasize the need for a biopsy and DIF in all of them. The list of medications implicated in DI-LAD development has grown in recent years, but one has to be cautious in establishing causality. The use of algorithms to estimate the probability of ADR can aid in establishing a diagnosis.
